# Specific Factors Associated with Iatrogenic Withdrawal Syndrome in Children After Cardiac Surgery

**DOI:** 10.3390/children13101307

**Published:** 2026-09-26

**Authors:** Ida Hüners, Sophia Schumann, Gerhard Schön, Daniel Biermann, Jonas Denecke, Urda Gottschalk, Peer P. Jürgensen, Lena C. Siebel, Friederike Jess, Sinno H. P. Simons, Carolin Gleitze-Nolting, Henning Carstens, Michael Hübler, Rainer G. Kozlik-Feldmann, Irwin K. M. Reiss, Sebastian H. Harms

**Affiliations:** 1 Department of Congenital and Pediatric Cardiac Surgery, Children’s Heart Clinic, University Heart & Vascular Center, University Medical Center Hamburg-Eppendorf, 20246 Hamburg, Germany; i.hueners@uke.de (I.H.); d.biermann@uke.de (D.B.); h.carstens@uke.de (H.C.); m.huebler@uke.de (M.H.); 2 Department of Pediatric Cardiology, Children’s Heart Clinic, University Heart & Vascular Center, University Medical Center Hamburg-Eppendorf, 20246 Hamburg, Germany; sophia.schumann@stud.uke.uni-hamburg.de (S.S.); u.gottschalk@uke.de (U.G.); p.juergensen@uke.de (P.P.J.); l.siebel@uke.de (L.C.S.); f.jess@uke.de (F.J.); c.gleitze-nolting@uke.de (C.G.-N.); r.kozlik-feldmann@uke.de (R.G.K.-F.); 3 Institute of Medical Biometry and Epidemiology, University Medical Center Hamburg-Eppendorf, 20246 Hamburg, Germany; g.schoen@uke.de; 4 Section Pediatric Neurology, Department of Pediatrics, Center for Obstetrics and Pediatrics, University Medical Center Hamburg-Eppendorf, 20246 Hamburg, Germany; j.denecke@uke.de; 5 Department of Pediatric and Neonatal Intensive Care, Division of Neonatology, Erasmus University Medical Center, 3015 GD Rotterdam, The Netherlands; s.simons@erasmusmc.nl (S.H.P.S.); i.reiss@erasmusmc.nl (I.K.M.R.); 6 Section Neonatology and Pediatric Intensive Care Medicine, Department of Pediatrics, Center for Obstetrics and Pediatrics, University Medical Center Hamburg-Eppendorf, 20246 Hamburg, Germany; i.reiss@uke.de

**Keywords:** iatrogenic withdrawal syndrome, pediatric cardiac intensive care, cardiac surgery, sedation weaning

## Abstract

**Highlights:**

**What are the main findings?**

• Iatrogenic withdrawal syndrome (IWS) was highly frequent in children after cardiac surgery (46.3%, 95% CI 40.6–52.0), and univentricular physiology, prolonged invasive mechanical ventilation, and suspected postoperative infection were independently associated with this occurrence.
• Mechanically ventilated children after cardiac surgery, who were sedated with midazolam or received prolonged analgesia with remifentanil, showed substantially higher, though not statistically significant, odds of IWS after adjustment for age, ventilation time, surgical complexity, univentricular physiology, and suspected postoperative infection.

**What are the implications of the main findings?**

• As IWS is highly frequent, systematic WAT-1 screening and structured sedation and weaning protocols should be the standard of care in children after cardiac surgery.
• Children with congenital heart disease, in particular those with univentricular physiology, may warrant individualized sedation strategies. Midazolam and prolonged remifentanil infusion showed substantially higher, though not significant, estimates and therefore warrant prospective investigation.

**Abstract:**

**Background/Objectives**: Iatrogenic withdrawal syndrome (IWS) is common but underrecognized in critically ill children, and data from pediatric cardiac intensive care units (pCICUs) remain limited. This study determined the frequency of IWS after cardiac surgery and the factors associated with it. **Methods**: Retrospective, single-center cohort study of all pediatric patients admitted to a pCICU after cardiac surgery over 15 months. IWS screening was performed using the Withdrawal Assessment Tool-1 (WAT-1) at least three times daily post-extubation. IWS was defined as at least one WAT-1 score of 3 or higher. A prespecified multivariable logistic regression model with ten perioperative variables was fitted in the complete-case population. A subgroup analysis in ventilated children examined sedative and analgesic exposure, including the grouped duration of remifentanil infusion, in an adjusted model. **Results**: Of 311 patients, 294 (94.5%) were screened, and IWS occurred in 136 patients (46.3%, 95% CI 40.6–52.0). In the complete-case population (*n* = 286), univentricular physiology (OR 2.51, *p* = 0.044), suspected postoperative infection (OR 5.28, *p* = 0.023), and invasive ventilation for 10–100 h (OR 2.75, *p* = 0.021) or beyond 100 h (OR 5.29, *p* = 0.003) were independently associated with IWS, whereas postoperative lactatemia (>2 mmol/L for >6 h) was associated with lower odds (OR 0.39, *p* = 0.039). In mechanically ventilated children (*n* = 162), after adjustment, no sedative or analgesic exposure was significantly associated with IWS, while odds were substantially higher in patients receiving remifentanil beyond 48 h (OR 2.64, *p* = 0.114) and midazolam (OR 3.95, *p* = 0.057). **Conclusions**: In our institution, IWS was highly frequent after pediatric cardiac surgery, in particular in patients with univentricular physiology. In sedated patients, midazolam and prolonged remifentanil showed substantially higher, though not statistically significant, odds of developing IWS. These hypothesis-generating associations require prospective confirmation.

## 1. Introduction

Children undergoing open cardiac surgery with or without cardiopulmonary bypass (CPB) constitute a particularly vulnerable group in the pediatric cardiac intensive care unit (pCICU), often requiring prolonged administration of opioids and benzodiazepines [1]. Prolonged exposure and its abrupt discontinuation or rapid weaning are linked to the development of iatrogenic withdrawal syndrome (IWS) [2], a variable constellation of neurobehavioral (e.g., agitation, tremor, insomnia), gastrointestinal (e.g., vomiting, diarrhea), and autonomic symptoms (e.g., tachycardia, hypertension, sweating). IWS is associated with significant morbidity in critically ill children [3], including postoperative delirium (PD) [4], the duration of which is linked to long-term neurological outcomes in adults [5]. In children with congenital heart disease (CHD), PD has recently been linked to neurodevelopmental outcomes [6], of particular interest, as almost 50% of CHD patients suffer from neurodevelopmental delays [7]. Yet only a few studies have investigated IWS prevalence and risk factors in this population, and knowledge is derived from combined pediatric intensive care unit (PICU) cohorts. We hypothesize that the individual physiology of CHD patients, CPB-induced systemic inflammation, low cardiac output (LCO), and the resulting hepatorenal impairment of drug clearance modify both sedative pharmacokinetics and central nervous system vulnerability, thereby shaping an IWS risk profile distinct from that of the general PICU population.

Reported IWS prevalence varies widely across PICU cohorts, reflecting differences not only in sedative exposure but also in the presence and rigor of standardized weaning protocols [8]. In a large pediatric multicenter cohort study on acute respiratory failure (RESTORE study), 47% of children sedated for five or more days developed IWS, with a higher risk in younger children and those with preexisting cognitive impairment or exposure to multiple sedative classes [9]. In mixed PICU cohort studies, in which almost half of the cases had undergone cardiac surgery, IWS occurred in 37% [10]. The Society of Critical Care Medicine (SCCM) therefore recommends validated assessment tools, of which the Withdrawal Assessment Tool (WAT-1) and the Sophia Observation Withdrawal Symptoms Scale (SOS) are the most widely applied [11]. Both demonstrate good sensitivity and specificity for IWS detection, but neither differentiates between opioid and benzodiazepine withdrawal [12,13]. Their routine use enables standardized diagnosis, facilitates early intervention, and supports evidence-based weaning protocols, thereby reducing both the prevalence and severity of IWS in PICU and pCICU patients [8].

In PICU populations, cumulative total dose and infusion duration are consistent predictors of IWS [14], although their relationship with IWS risk is not strictly linear [15]. One of the most relevant factors in IWS prevention is maintaining an adequate, individually titrated depth of sedation: in a randomized controlled trial, protocolized sedation using the COMFORT Behavior Scale (COMFORT-B) alone reduced the duration of mechanical ventilation, cumulative sedative exposure, and sedation-related adverse events [16]. Nevertheless, plasma concentrations of sedatives vary widely despite identical dosing due to interindividual differences in metabolism and elimination [17]. In children after cardiac surgery, systemic inflammation, LCO, and frequent renal and hepatic dysfunction may further impair drug clearance, promoting drug accumulation and increasing the risk of IWS during sedation weaning [18,19,20,21].

Therefore, investigating factors associated with IWS specifically in the pCICU cohort is necessary to develop individualized sedation and weaning protocols, as is research on the impact of IWS on neurodevelopmental outcomes. In addition, over the past decade, major changes in the pCICU setting have emerged that are relevant to IWS. Fast-track protocols have had a major impact on ventilation duration, length of stay, and cumulative drug application in the pCICU [22], supported by new analgesics such as remifentanil, a rapidly metabolized, organ-independent agent. To our knowledge, no study has used SOS or WAT-1 to assess IWS after remifentanil infusion, although German clinical guidelines [23] recommend remifentanil for up to 72 h, based on safety data from adult studies [24,25].

## 2. Materials and Methods

### 2.1. Study Design and Patient Characteristics

This retrospective, single-center observational study was conducted over 15 months (March 2023 to June 2024) at a tertiary university medical center to determine the frequency and factors associated with IWS in pediatric patients after cardiac surgery. Term neonates and children up to 18 years of age admitted to the pCICU following cardiac surgery with or without CPB were included. Patients undergoing minor interventions or non-cardiac procedures (e.g., central venous catheter placement, drainage placement, cardioversion, etc.) with only short-term sedation, defined as single-dose or procedural sedation without continuous sedative or analgesic infusion, were excluded. Patient selection is shown in Supplementary Figure S1. The study adhered to the Declaration of Helsinki, and, given its retrospective nature, the Hamburg Medical Association Ethics Committee waived the requirement for informed consent (2023-300407-WF) for the study titled “Delirium Prevalence and Risk Factors in Children after Cardiac Surgery” on 9 November 2023. The approval covers the present analysis of IWS in the same cohort. Postoperative delirium and IWS were screened concurrently using separate validated instruments and analyzed and reported separately [4]. According to our standard protocol, postoperative sedation is primarily achieved with dexmedetomidine (0.5–1.4 µg/kg/h) in nearly all patients; therefore, it could not be evaluated as a predictor because there was no unexposed comparator. Additional continuous ketamine or midazolam is avoided whenever possible according to current guideline recommendations [11]. Muscle relaxation with rocuronium (0.6–1.0 mg/kg/h) is reserved for high-pressure ventilation settings to reduce patient-ventilator dyssynchrony. First-line analgesia consists of single-dose opioids (morphine or piritramide 0.1 mg/kg) at extubation or remifentanil infusion (0.05–2.0 µg/kg/min) when short-term ventilation is anticipated. In more complex courses, increasing tolerance may necessitate switching to morphine (30–100 µg/kg/h) or sufentanil (0.3–1.3 µg/kg/h) infusions. Weaning protocols after prolonged exposure are standardized and guided by COMFORT-B and WAT-1. Supplementary Figure S2 provides further details about our decision and treatment algorithm.

### 2.2. Screening Protocol and Assessment Instruments

IWS was assessed with the WAT-1 [26], a clinician-administered instrument developed to identify and quantify IWS in pediatric patients after prolonged exposure to opioids and/or benzodiazepines in intensive care. It consists of 11 items, generating a total score of 0–12. Assessments were performed by trained doctors and nurses in teams at three fixed times per day, corresponding to an interval of approximately eight hours, in all patients who were sufficiently awake to be scored, defined as a Richmond Agitation and Sedation Scale (RASS) of −2 or higher. Opioids and sedatives were tapered according to the institutional weaning protocol, in which reduction steps are themselves guided by the WAT-1 score (Supplementary Figure S3).

PD was screened routinely and concurrently using the Cornell Assessment of Pediatric Delirium (CAPD) and was analyzed separately in a companion study of the same cohort [4]. IWS (WAT-1) and PD (CAPD) were therefore captured with two distinct, validated instruments. We acknowledge that behavioral items overlap across these tools [27]. However, in our protocol, the two tools were administered independently at each assessment, thereby limiting misattribution between the two syndromes.

### 2.3. Outcome Definition

The outcome of this study was the occurrence of IWS, defined a priori as at least one WAT-1 score of 3 or higher recorded during the postoperative pCICU stay. A single positive assessment was sufficient to classify a patient as having IWS, and repeated positive scores were not required. The observation period began with the first post-extubation assessment and ended with discharge from the pCICU. Screening was not continued after transfer to the peripheral ward. The reported frequency therefore denotes the cumulative occurrence of at least one positive assessment during the postoperative pCICU stay rather than a point prevalence, and IWS with a later onset after transfer would not have been captured. This retrospective dataset cannot establish the temporal relationship between exposure and outcome with certainty. All postoperative variables are therefore reported as factors associated with IWS rather than as predictors, and reverse causation cannot be excluded for variables that may develop after the first positive assessment.

### 2.4. Risk Factor Selection and Categorization

Based on existing literature for pediatric and adult populations in critical care medicine, we selected candidate variables and grouped them into the following categories:

- Patient Demographics

Age categories: (1) neonates (0–28 days), (2) infants (>28 days–1 year), (3) toddlers/preschool children (>1–6 years), and (4) school-aged children/adolescents (>6–18 years).

- Surgical Factors

Surgical complexity: Risk Adjustment for Congenital Heart Surgery 1 (RACHS-1) score, presence of univentricular physiology, and CPB duration.

- Physiological Derangements and Low Cardiac Output

Postoperative desaturation: SpO_2_ < 92% for > 6 h. This threshold was not adjusted for the lower baseline saturations expected in patients with cyanotic or univentricular physiology and therefore cannot be interpreted uniformly across the cohort. Because of this limitation, the variable was recorded but not included in the principal multivariable model. Hyperglycemia: blood glucose > 200 mg/dL; lactatemia: lactate > 2 mmol/L for > 6 h; lactatemia subgroup used: lactatemia plus avDO2 > 40% plus core-to-peripheral temperature gradient (ΔT) > 4 °C to distinguish type A (low cardiac output (LCO)-related) from metabolic type B lactatemia (lactatemia plus hyperglycemia) [28].

- Inflammatory Markers and Infection

Postoperative fever: >38.5 °C for >3 h; clinical signs of suspected postoperative infection: postoperative fever plus secondary C-reactive protein elevation (not related to a second surgical intervention) and/or positive microbiology and/or clinical decision to restart antibiotic treatment. A patient was classified as positive if at least one criterion was met (because postoperative fever and C-reactive protein elevation are common after CPB, this composite identifies suspected rather than microbiologically proven infection). Hydrocortisone was administered for hemodynamic support in the context of systemic inflammatory response syndrome (SIRS) at the treating team’s discretion. Formal assessment of adrenal insufficiency by ACTH stimulation testing was not routinely performed before initiation.

- Ventilation and Sedation Parameters

Ventilation: on-table extubation (OTE), fast-track (≤10 h), short-term (>10–100 h), long-term (>100 h). Patients who were ventilated beyond the operating room had continuous sedative infusions recorded: remifentanil, morphine, sufentanil, and midazolam. The use of dexmedetomidine was not suitable for statistical analysis because it was administered to almost every patient per our protocol (Supplementary Figure S2); WAT-1 is not validated for dexmedetomidine withdrawal, and evidence linking it to withdrawal remains limited, even if it is the only sedative used [29]. Ketamine-related withdrawal was not analyzed as the items tested by WAT-1 seem to be insensitive to the symptoms described [30].

### 2.5. Data Collection and Statistical Analysis

Demographic and clinical data were extracted from the patient data management system (Intensive Care Manager (ICM), version 13.02; Drägerwerk, Lübeck, Germany) using ICMiq (version 1.5.0) and were pseudonymized by an independent trustee before analysis.

Categorical variables were described using frequencies and proportions, and continuous variables using means with standard deviations. Group comparisons used *t*-tests for continuous and chi-square tests for categorical data. Confidence intervals are reported for the frequency of IWS. Candidate variables for the multivariable models were selected a priori based on a literature review and entered together. No data-driven variable selection was performed. Therefore, the principal model was prespecified as the primary model. We used multivariable logistic regression to model IWS as a function of various independent variables and report odds ratios, 95% confidence intervals, and *p*-values for these variables. Each model is displayed graphically as a forest plot. The principal model explained the probability of IWS by ten variables recorded during the post-operative pCICU stay: age category (four levels, neonates as reference), RACHS-1 score (metric, 1–6), univentricular physiology, metabolic derailment, cardiopulmonary bypass time (per 30-min interval), lactatemia, ventilation class (four levels, OTE as reference), fever, suspected infection, and hydrocortisone administration. All models were fitted as complete-case analyses. Of the 294 patients with a valid WAT-1 result, 286 had complete information on all ten model variables and constituted the population of the principal model. The subgroup analysis in ventilated children examined exposure to morphine, sufentanil, and midazolam (each as exposure versus non-exposure) together with the grouped total duration of remifentanil infusion, entered as a three-level categorical variable with an infusion of up to 24 h as the reference category (>24 to 48 h; more than 48 h). This model was adjusted for age category, ventilation class (three levels, fast track as reference), RACHS-1 score, univentricular physiology, and suspected infection. The corresponding figure legend provides the complete list of covariates for each model, along with the number of patients and IWS events. Because the principal model was prespecified as the primary model, no adjustment for multiple comparisons was applied. The subgroup analyses reported in Section 3.5 are exploratory, and their *p*-values are not adjusted. We tested for possible multicollinearity selectively among variables using Kendall’s tau-c for ordinal variables. For the two clinically most closely related variables of the principal model, suspected postoperative infection and ventilation class, the ordinal association was weak (tau-c = 0.158), indicating that they contribute distinct information to the model. All analyses and figures were calculated using the statistical software R, version 4.6.1 (R Core Team. R: A Language and Environment for Statistical Computing. R Foundation for Statistical Computing, Vienna, Austria, 2026), and the R package effects, version 4.2.5.

To assess whether the exclusion of patients without a WAT-1 result introduced selection bias, the 17 patients without any IWS scoring were compared with the complete-case population of the principal model (*n* = 286; Supplementary Table S1) and, separately, with the population of the ventilated subgroup model (*n* = 162; Supplementary Table S2) with respect to their baseline and perioperative characteristics. The eight patients who had a valid WAT-1 result but incomplete information on one or more model variables were not included in the complete-case population because all models were fitted as complete-case analyses.

### 2.6. Sample Size Calculation

The primary goal of the study was to estimate the frequency of IWS using the WAT-1 screening tool. A 95% confidence interval not greater than ±6% was considered clinically relevant. We assumed the prevalence to be between 40% and 50%. Therefore, a 40% prevalence would require *n* = 271 cases, and a 50% prevalence would require *n* = 281 cases. We planned conservatively and decided on a sample size of *n* = 281 cases. The sample size was calculated to estimate the frequency of IWS, which was the primary objective of the study. It was not designed to ensure adequate power for the multivariable analyses. The adequacy of the sample for those models is addressed in the Limitations.

## 3. Results

### 3.1. Study Population and Frequency of IWS

From March 2023 to June 2024, 349 patients aged 0–18 years were admitted to our pCICU. After excluding 38 patients (10.9%) undergoing minor interventions and non-cardiac procedures with only short-term sedation, 311 patients who underwent cardiac surgery, with or without CPB, were included (Supplementary Figure S1). A valid WAT-1 result was available for 294 of the 311 patients, corresponding to a screening rate of 94.5%. In 17 patients, no WAT-1 assessment was documented. These 294 patients are referred to as the screened population throughout the manuscript. IWS occurred in 136 of them (46.3%, 95% CI 40.6–52.0). Complete information on all ten variables of the principal model was available for 286 of the 294 screened patients. The remaining eight patients were excluded from the multivariable analyses, which were fitted as complete-case analyses. These 286 patients are referred to as the complete-case population. Within this population, 131 patients (45.8%, 95% CI 40.1–51.6) had IWS. Baseline and perioperative characteristics of the complete-case population by IWS status are shown in Table 1.

### 3.2. Perioperative Factors Associated with IWS

Perioperative factors associated with IWS are summarized in Figure 1. Age category, RACHS-1 score, cardiopulmonary bypass time (CPBT), metabolic derailment, fever, and hydrocortisone administration were not significantly associated with IWS in the multivariable model. Univentricular physiology was associated with higher odds of IWS (OR 2.51, 95% CI 1.04–6.27, *p* = 0.044), as were clinical signs of suspected postoperative infection (OR 5.28, 95% CI 1.39–26.51, *p* = 0.023). Compared with OTE, both short-term (10–100 h) and long-term (>100 h) invasive mechanical ventilation were associated with higher odds of IWS (OR 2.75, 95% CI 1.17–6.61, *p* = 0.021 and OR 5.29, 95% CI 1.80–16.26, *p* = 0.003, respectively), whereas fast-track extubation was not (OR 1.53, 95% CI 0.70–3.34, *p* = 0.285). Lactatemia was associated with lower odds of IWS (OR 0.39, 95% CI 0.15–0.94, *p* = 0.039).

### 3.3. Sedative and Analgesic Exposure in Ventilated Children

Of the 311 patients, 187 did not receive OTE but were extubated via the fast-track protocol or required short- or long-term mechanical ventilation. A valid WAT-1 result and complete information on all model variables were available for 162 of them, and this population forms the basis of the analysis shown in Figure 2. IWS occurred in 93 of these 162 patients (57.4%, 95% CI 49.7–64.8). Exposure to morphine, sufentanil, and midazolam, along with the grouped total duration of remifentanil infusion, were examined together in a single model, adjusted for age category, ventilation class, RACHS-1 score, univentricular physiology, and suspected infection.

None of the individual sedative or analgesic exposures reached statistical significance. Relative to an infusion of up to 24 h, a remifentanil infusion of more than 24 and up to 48 h was not associated with IWS (OR 0.80, 95% CI 0.23–2.72, *p* = 0.717). The same applied to morphine (OR 0.56, 95% CI 0.15–1.93, *p* = 0.361) and sufentanil (OR 0.79, 95% CI 0.16–3.69, *p* = 0.762). An infusion of remifentanil beyond 48 h showed substantially higher, though not statistically significant, odds (OR 2.64, 95% CI 0.81–9.14, *p* = 0.114), while the estimate for midazolam was the largest among the agents but did not reach statistical significance either (OR 3.95, 95% CI 1.01–17.90, *p* = 0.057).

Among the covariates of the same model, infant age (OR 3.98, 95% CI 1.50–11.27, *p* = 0.007) and univentricular physiology (OR 5.31, 95% CI 1.44–23.75, *p* = 0.018) were associated with IWS, whereas RACHS-1 score (OR 1.08, 95% CI 0.70–1.68, *p* = 0.743), suspected infection (OR 3.17, 95% CI 0.75–18.10, *p* = 0.146), short-term ventilation (OR 1.42, 95% CI 0.50–4.13, *p* = 0.516) and long-term ventilation (OR 1.13, 95% CI 0.20–6.19, *p* = 0.884) were not. Within this ventilated subgroup, patient-related factors rather than the individual sedative or analgesic agent were associated with the occurrence of IWS.

### 3.4. Sensitivity Analysis

The 17 patients without any WAT-1 assessment did not differ from the complete-case population of the principal model in any recorded characteristic except ventilation class: they had more often received long-term ventilation (41.2% versus 17.1%, *p* = 0.030), whereas age, RACHS-1 score, univentricular physiology, cardiopulmonary bypass time, metabolic derailment, lactatemia, fever, suspected infection and hydrocortisone administration were comparable (Supplementary Table S1). The same comparison against the population of the ventilated subgroup model showed no relevant difference (Supplementary Table S2).

### 3.5. Exploratory Subgroup Analyses

Two subgroup analyses were performed to explore whether the two variables that had shown an association in the principal model could be characterized further. Neither analysis produced a statistically significant result, and both are therefore reported as exploratory and are presented in the Supplementary Material.

Among the 77 patients with documented lactatemia and a valid WAT-1 result, none of the three lactatemia subtypes was associated with IWS (low cardiac output OR 2.41, 95% CI 0.78–7.91, *p* = 0.134; metabolic derailment OR 0.52, 95% CI 0.02–5.09, *p* = 0.605; lactatemia without either criterion OR 0.33, 95% CI 0.01–4.42, *p* = 0.434; Supplementary Figure S4).

Among the 49 patients with univentricular physiology, none of the palliation stages differed significantly from biventricular repair (Stage 1 OR 1.29, 95% CI 0.13–13.07, *p* = 0.822; Glenn OR 12.00, 95% CI 0.80–353.22, *p* = 0.085; Fontan OR 1.00, 95% CI 0.09–10.76, *p* = 1.000; Supplementary Figure S5). All confidence intervals span more than one order of magnitude, and no definitive conclusion can be drawn from either analysis.

## 4. Discussion

This retrospective study represents one of the first large cohorts to comprehensively investigate factors associated with IWS in children after cardiac surgery for congenital heart disease. The IWS frequency of 46.3% in our cohort exceeded that of a comparable pCICU study [31] but matched PICU studies [9]. Our findings identify clinical and pharmacologic factors associated with IWS in children after cardiac surgery. Rather than establishing mechanistic causation, our findings generate hypotheses regarding the pathways and sedation-weaning practices warranting prospective evaluation in this vulnerable population.

No significant age-dependent variation in IWS was observed across pediatric age groups. Infants and toddlers had numerically higher odds ratios than neonates, similar to general PICU populations [9] but slightly different from previous pCICU data [31]. Of interest, after adjusting for the variables in our multivariable model, neither CPBT nor cardiac disease severity was associated with IWS. At the same time, patients with univentricular physiology were significantly more affected. To our knowledge, this association has not been reported previously in this population.

Prolonged invasive mechanical ventilation was associated with higher odds of IWS in our cohort. This is consistent with, though it does not prove, a cumulative-exposure hypothesis, since ventilation time is an imperfect surrogate for cumulative drug dose and may vary considerably between patients ventilated for similar periods [14]. Additional use of amplitude-integrated electroencephalography (aEEG) in the operating room helps optimize sedation depth [32] and, when combined with protocolized behavioral assessment in the PICU, might further improve these clinical outcomes by reducing overall cumulative dose [33].

In the adjusted analysis of ventilated children, the grouped total duration of remifentanil infusion was not associated with IWS: relative to an infusion of up to 24 h, neither an infusion of more than 24 and up to 48 h nor one exceeding 48 h reached statistical significance, but the estimate for the longest exposure category was OR 2.64 (95% CI 0.81–9.14, *p* = 0.114). Remifentanil is therefore not supported as an independent correlate of IWS in our data. A plausible reading is that patients receiving prolonged remifentanil infusions also had longer respiratory support, greater illness severity, and greater cumulative exposure to other agents, and that these patient- and course-related factors, rather than the agent itself, account for the crude association. Cumulative per-agent doses were unavailable, so we cannot exclude a dose-dependent effect below the resolution of our exposure categories. This question requires a prospective design with pharmacokinetic data. To our knowledge, this represents the first large pediatric intensive care cohort using remifentanil for up to 72 h postoperatively, and there is limited evidence that prolonged remifentanil infusion is inferior to morphine infusion for IWS [34].

The ester metabolism of remifentanil results in extremely rapid plasma clearance [35], so that even brief interruptions in infusion produce profound, sudden reductions in plasma concentration. This pharmacokinetic property, distinct from the hepatic and renal metabolism [36] that governs morphine and other opioids, renders remifentanil peculiarly vulnerable to precipitous concentration gradients and would be a plausible mechanism if such an association were confirmed in a study with cumulative dosing data.

In our cohort, as per protocol (Supplementary Figure S2), remifentanil infusions were typically discontinued upon completion of surgery or shortly thereafter, with morphine or other long-acting opioids initiated 30–60 min before the anticipated end of remifentanil. The narrow temporal window of overlap may inadequately attenuate the steeply declining remifentanil plasma curve, leaving a pharmacodynamic “gap” for withdrawal symptoms to emerge. These observations generate the hypothesis that stricter duration limits for prolonged remifentanil infusion, or transition protocols with an extended overlap with long-acting opioids, could reduce IWS in this population. This hypothesis cannot be tested with the present data. A prospective study specifically designed to compare remifentanil transition strategies would be invaluable for identifying optimal weaning protocols.

Midazolam showed the largest point estimate among the agents examined but did not reach statistical significance after adjustment (OR 3.95, 95% CI 1.01–17.90, *p* = 0.057), so no independent association can be claimed. Though we interpret this association with caution, midazolam at our institution is typically administered as a last-in-line sedation, per our protocol (Supplementary Figure S2), for inadequately sedated, hemodynamically fragile patients, thereby representing patients with a higher cumulative dose of all agents and higher postoperative disease severity.

Clinical signs of suspected postoperative infection showed the strongest association with IWS in the principal model, although the estimate is imprecise (95% CI 1.39–26.51, 28 affected patients) and the composite definition identifies suspected rather than microbiologically proven infection. A mechanistic link between infectious complications and opioid–benzodiazepine withdrawal is plausible but is not demonstrated by these data. Cross-tabulation of suspected postoperative infection against ventilation class showed only a weak ordinal association (Kendall’s tau-c = 0.158), indicating that the two variables contribute distinct information to the model rather than duplicating one another. Postoperative infections frequently precipitate hepatorenal dysfunction, altered drug distribution volumes secondary to capillary leak and hypoalbuminemia, and markedly increased variability in plasma concentrations of opioids and benzodiazepines [37], thereby making adequate sedation and weaning more difficult. Further investigation, including plasma drug monitoring, could address this question. Additionally, systemic infection activates neuroinflammatory cascades that enhance neuronal hyperexcitability, promote analgesic tolerance, and amplify withdrawal severity through increased cumulative drug requirements [38].

Morphine itself contributes to neuroinflammatory priming, potentially establishing a vicious cycle whereby morphine-induced microgliosis predisposes to more severe infection-related neuroinflammation and, consequently, more pronounced withdrawal manifestations [39]. Preliminary preclinical evidence suggests that anti-inflammatory therapeutic strategies may attenuate opioid withdrawal severity in model systems [40], though human translation remains preliminary.

The lack of association with hydrocortisone is particularly interesting given our institution’s routine perioperative anti-inflammatory practice. This may reflect effective attenuation of fever through postoperative pharmacologic (acetaminophen, metamizole) and temperature-control strategies, thereby masking the true relationship between inflammatory activation and IWS via fever suppression.

In the principal model, lactatemia was associated with lower odds of IWS (OR 0.39, 95% CI 0.15–0.94, *p* = 0.039). We regard this observation as exploratory. The subgroup analysis intended to distinguish low-cardiac-output-related from metabolic lactatemia produced no significant estimate (Supplementary Figure S4) and does not allow a definitive conclusion. Notably, in the companion analysis of postoperative delirium in the same cohort, lactatemia was associated with significantly higher odds of delirium [4], a finding in the opposite direction. One speculative explanation is that hyperlactatemia may in part reflect impaired hepatic clearance rather than tissue hypoperfusion [41,42], which could be accompanied by higher plasma concentrations of sedatives and opioids [18] and thereby attenuate withdrawal symptoms. Testing this hypothesis would require plasma drug concentrations and cumulative dosing data, which were not available. Prospective studies combining WAT-1 screening with therapeutic drug monitoring of sedatives and opioids would be required to test it.

This study has several limitations. The most important concern is the assessment of drug exposure. Per-agent cumulative doses were not available in this retrospective single-center dataset, so exposure had to be analyzed categorically, with the duration of invasive mechanical ventilation serving only as an imperfect surrogate for cumulative drug exposure. The adjusted analysis of the ventilated subgroup combines four drug variables with five adjustment variables in a comparatively small population, which yields wide confidence intervals. A clinically relevant association of an individual agent therefore cannot be excluded on the basis of these data.

The ascertainment of the outcome has limitations of its own. All models were fitted as complete-case analyses: 17 of 311 patients had no WAT-1 assessment and a further 8 lacked information on at least one adjustment variable, so that the principal model rests on 286 patients. A comparison of the non-screened patients with the complete-case population is provided in Supplementary Tables S1 and S2. The two groups were comparable in all recorded characteristics except ventilation class: patients without a WAT-1 assessment had more often received long-term ventilation (41.2% versus 17.1%, *p* = 0.030). Exclusion of these patients may therefore have led to an underestimation of the reported frequency of IWS. Because IWS status is unknown in this group, however, the exact direction and magnitude of the resulting bias cannot be determined. Screening was not continued after transfer from the pCICU to the peripheral ward, so withdrawal with a later onset may have remained undetected. This is particularly relevant in younger patients, in whom reduced drug clearance may delay the onset of symptoms. In addition, the WAT-1 has limited sensitivity for withdrawal associated with alpha-2 agonists and ketamine, which may therefore not have been captured reliably.

Several variable definitions require caution. Postoperative desaturation was defined without adjustment for the lower baseline saturations expected in cyanotic or univentricular physiology and cannot be interpreted uniformly across the cohort. Because this variable and univentricular physiology are clinically related, the estimate for univentricular physiology should be interpreted in light of this relationship. More generally, several candidate variables are physiologically interdependent, and their associations cannot be fully disentangled from duration of ventilation and from one another.

Estimates based on small numbers of patients should not be over-interpreted. The exploratory subgroup analyses of lactatemia subtypes (*n* = 77) and univentricular palliation stage (*n* = 49) produced no significant estimates and are reported in the Supplementary Material (Figures S4 and S5) for completeness only. The estimate for suspected postoperative infection is based on 28 affected patients. The correspondingly wide confidence intervals are compatible with a broad range of underlying effects, and no conclusion about risk should be drawn from these analyses.

Two further constraints limit what can be inferred. Because several postoperative variables develop during the pCICU stay and their temporal sequence relative to the first positive WAT-1 assessment could not be established with certainty, reverse causation cannot be excluded, and no causal interpretation of any association reported here is intended. The study is also monocentric. Sedation practices and drug regimens differ between institutions, and the use of remifentanil and midazolam at our center may not be representative. Other drug combinations could yield a different frequency of IWS. Multicenter studies evaluating alternative regimens are therefore needed.

## 5. Conclusions

In this retrospective single-center cohort, IWS was frequent in children recovering from cardiac surgery in a pCICU. Univentricular physiology, prolonged invasive mechanical ventilation, and clinical signs of suspected postoperative infection were significantly associated with higher odds of IWS, whereas lactatemia was associated with lower odds. In the adjusted analysis of ventilated children, the point estimates for prolonged remifentanil infusion (OR 2.64) and for midazolam (OR 3.95) were substantially above 1 but did not reach statistical significance. These findings are observational and hypothesis-generating. They identify candidate targets for prospective evaluation rather than established causal risk factors.

We propose that future investigation in this population should prioritize individualized sedation and weaning protocols guided by pharmacokinetic and pharmacodynamic prediction models or direct evaluation of plasma concentrations. Hepatorenal dysfunction, infection, and individual pharmacogenetic variation may create wide interindividual differences in drug clearance in this specific population. Prospective studies examining alternative remifentanil transition strategies, the efficacy of extended overlap protocols with long-acting opioids, and the potential benefit of aEEG-based depth-of-anesthesia monitoring on pCICU could yield actionable interventions for this high-vulnerability population toward reducing IWS-related morbidity in pediatric cardiac critical care.

## Figures and Tables

**Figure 1:**
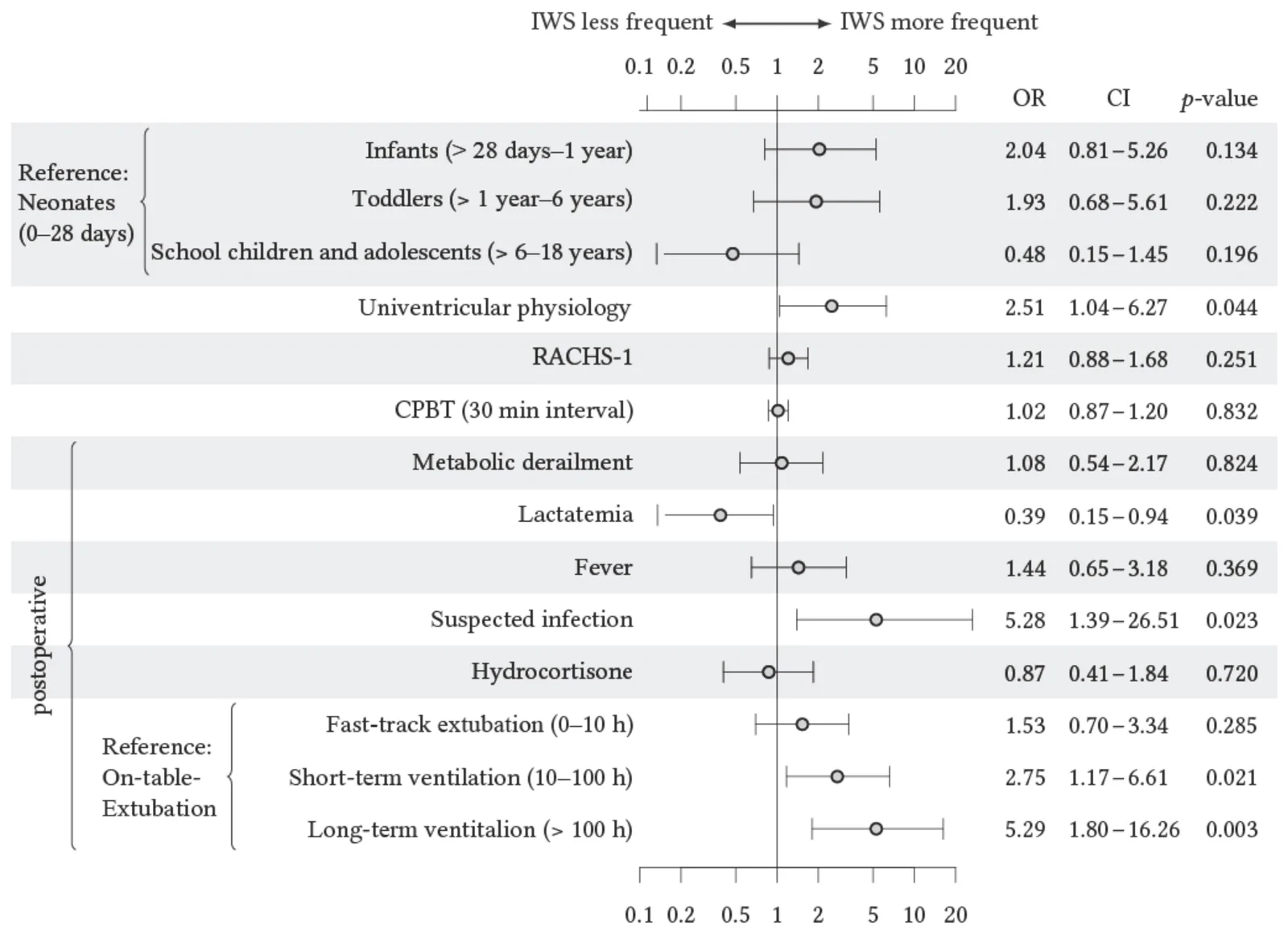
Multivariable logistic regression model for factors associated with IWS (complete-case population, *n* = 286, 131 IWS positive, ten model variables). Covariates: age category (reference: neonates, 0–28 days), RACHS-1 score, univentricular physiology, metabolic derailment, CPBT (per 30-min interval), lactatemia, ventilation class (reference: OTE), fever, suspected infection, and hydrocortisone administration. Odds ratios are shown with 95% confidence intervals and *p*-values. CPBT: cardiopulmonary bypass time; IWS: iatrogenic withdrawal syndrome; OTE: on-table extubation; RACHS-1: Risk Adjustment for Congenital Heart Surgery.

**Figure 2:**
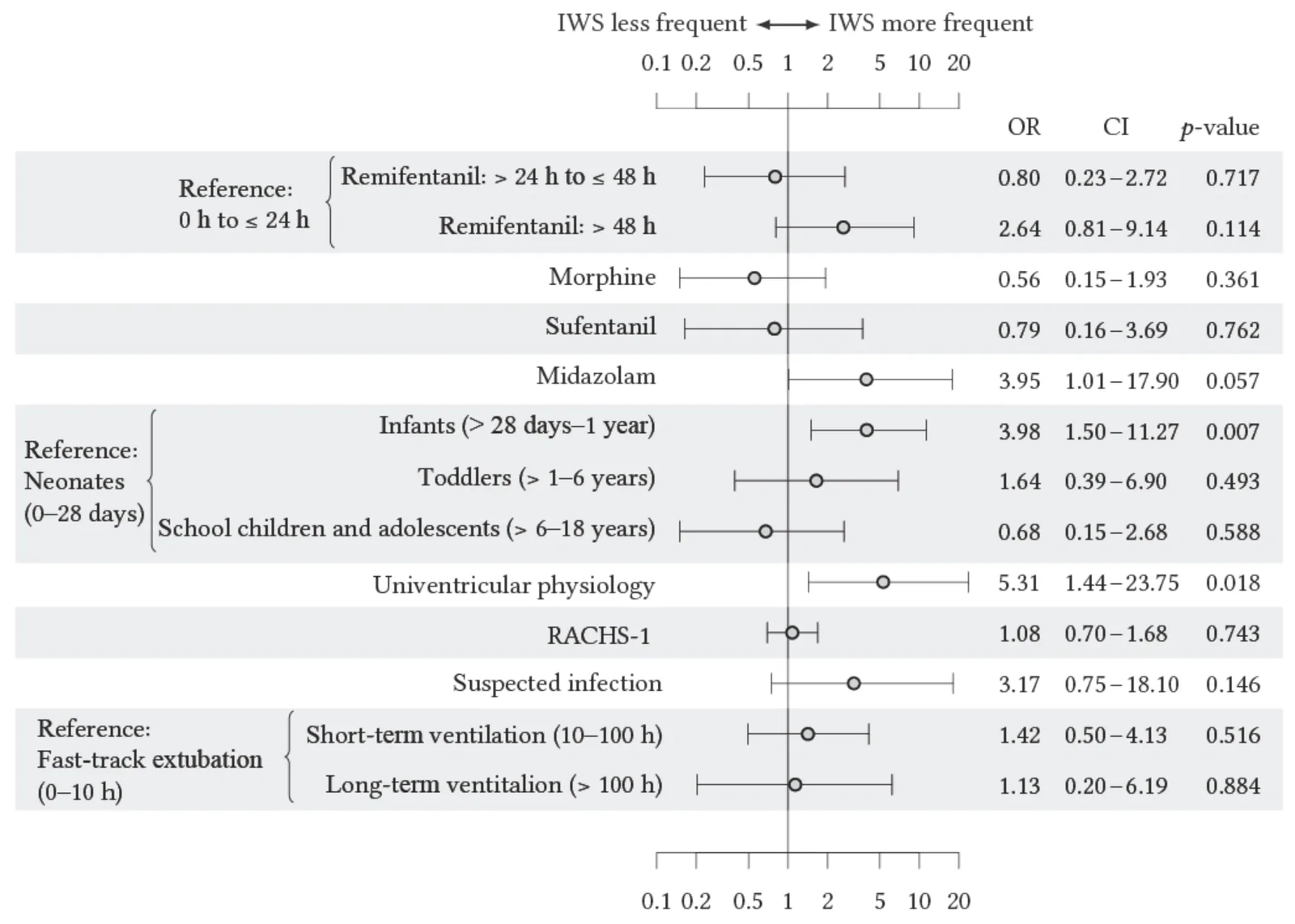
Multivariable logistic regression model for sedative and analgesic exposure and IWS in children who received fast-track extubation, short-term or long-term invasive mechanical ventilation (*n* = 162; 93 IWS positive). The total duration of remifentanil infusion was entered as a three-level categorical variable with an infusion of up to 24 h as the reference category. Morphine, sufentanil, and midazolam were entered as exposure versus non-exposure. The model was adjusted for age category (reference: neonates (0–28 days)), ventilation class (reference: fast-track extubation), RACHS-1 score, univentricular physiology, and suspected infection. Odds ratios are shown with 95% confidence intervals and *p*-values. IWS: Iatrogenic withdrawal syndrome; RACHS-1: Risk Adjustment for Congenital Heart Surgery.

**Table 1:** Demographic and clinical characteristics by occurrence of IWS in the complete-case population (*n* = 286). Data are *n* (%). *p*-values were derived from chi-square tests. CPBT: cardiopulmonary bypass time; IWS: iatrogenic withdrawal syndrome; pCICU: pediatric cardiac intensive care unit; RACHS-1: Risk Adjustment for Congenital Heart Surgery; WAT-1: Withdrawal Assessment Tool 1.

Characteristics	No. of Subjects (%)	No IWS (WAT-1 < 3) During pCICU Stay (%)	IWS (WAT-1 ≥ 3) During pCICU Stay (%)	*p* Value
*n*	286 (100.0)	155 (54.2)	131 (45.8)	
Age				<0.001
Neonates (0–28 days)	58 (20.3)	34 (21.9)	24 (18.3)	
Infants (>28 days–1 year)	116 (40.6)	45 (29.0)	71 (54.2)	
Toddlers (>1–6 years)	50 (17.5)	24 (15.5)	26 (19.8)	
School children and adolescents (>6–18 years)	62 (21.7)	52 (33.5)	10 (7.6)	
RACHS-1				0.022
1	34 (11.9)	24 (15.5)	10 (7.6)	
2	96 (33.6)	54 (34.8)	42 (32.1)	
3	106 (37.1)	58 (37.4)	48 (36.6)	
4	34 (11.9)	15 (9.7)	19 (14.5)	
5	1 (0.3)	1 (0.6)	0 (0.0)	
6	15 (5.2)	3 (1.9)	12 (9.2)	
Univentricular physiology				0.003
No	243 (85.0)	141 (91.0)	102 (77.9)	
Yes	43 (15.0)	14 (9.0)	29 (22.1)	
CPBT (minutes)				0.086
0–30	45 (15.7)	24 (15.5)	21 (16.0)	
>30–60	49 (17.1)	28 (18.1)	21 (16.0)	
>60–90	80 (28.0)	52 (33.5)	28 (21.4)	
>90–120	54 (18.9)	27 (17.4)	27 (20.6)	
>120	58 (20.3)	24 (15.5)	34 (26.0)	
Metabolic derailment				0.014
No	178 (62.2)	107 (69.0)	71 (54.2)	
Yes	108 (37.8)	48 (31.0)	60 (45.8)	
Postoperative desaturation				0.055
No	241 (84.3)	137 (88.4)	104 (79.4)	
Yes	45 (15.7)	18 (11.6)	27 (20.6)	
Lactatemia				0.579
No	215 (75.2)	114 (73.5)	101 (77.1)	
Yes	71 (24.8)	41 (26.5)	30 (22.9)	
Fever				<0.001
No	222 (77.6)	135 (87.1)	87 (66.4)	
Yes	64 (22.4)	20 (12.9)	44 (33.6)	
Suspected infection				<0.001
No	258 (90.2)	152 (98.1)	106 (80.9)	
Yes	28 (9.8)	3 (1.9)	25 (19.1)	
Hydrocortisone				0.004
No	194 (67.8)	117 (75.5)	77 (58.8)	
Yes	92 (32.2)	38 (24.5)	54 (41.2)	
Duration of mechanical ventilation (hours)				<0.001
On-table extubation	121 (42.3)	83 (53.5)	38 (29.0)	
Fast-track extubation (0–10)	48 (16.8)	27 (17.4)	21 (16.0)	
Short-term ventilation (10–100)	68 (23.8)	33 (21.3)	35 (26.7)	
Long-term ventilation (>100)	49 (17.1)	12 (7.7)	37 (28.2)	

## Data Availability

The raw data supporting the conclusions of this article will be made available by the corresponding author on request.

## Supplementary Materials

The following supporting information can be downloaded at https://www.mdpi.com/article/10.3390/children13101307/s1. Supplementary Figure S1: Flow-chart for patient selection; Supplementary Figure S2: Algorithm for perioperative analgosedation; Supplementary Figure S3: WAT-1-guided sedation-weaning algorithm; Supplementary Table S1: Sensitivity analysis: patients without any WAT-1 assessment compared with the complete-case population; Supplementary Table S2: Characteristics of the mechanically ventilated subgroup compared with the patients without any WAT-1 assessment; Supplementary Figure S4: Exploratory subgroup analysis of the 77 patients with documented lactatemia; Supplementary Figure S5: Exploratory subgroup analysis of the 49 patients with univentricular physiology.

## Abbreviations

The following abbreviations are used in this manuscript:

aEEG	Amplitude-integrated electroencephalography
CAPD	Cornell Assessment of Pediatric Delirium
CHD	Congenital heart disease
CI	Confidence interval
COMFORT-B	COMFORT Behavior Scale
CPB	Cardiopulmonary bypass
CPBT	Cardiopulmonary bypass time
IWS	Iatrogenic withdrawal syndrome
LCO	Low cardiac output
OR	Odds Ratio
OTE	On-table extubation
pCICU	Pediatric cardiac intensive care unit
PD	Postoperative delirium
PICU	Pediatric intensive care unit
RACHS-1	Risk Adjustment for Congenital Heart Surgery 1
RASS	Richmond Agitation and Sedation Scale
SCCM	Society of Critical Care Medicine
SIRS	Systemic inflammatory response syndrome
SOS	Sophia Observation Withdrawal Symptoms-scale
WAT-1	Withdrawal Assessment Tool-1
