# Supplementary material for: Specific Factors Associated with Iatrogenic Withdrawal Syndrome in Children After Cardiac Surgery
Source: Children (Basel). 2026 Sep 26;13(10):1307. doi: 10.3390/children13101307 (PMC13703752; doi:10.3390/children13101307)

Mapping a Specific Risk Profile for Iatrogenic Withdrawal Syndrome in Children after Cardiac Surgery

Supplemental Digital Content

Supplementary Figure S1: Flow chart of patient selection. Of 349 patients aged 0–18 years admitted to the pediatric cardiac intensive care unit between March 2023 and June 2024, 311 underwent cardiac surgery with or without CPB and formed the eligible cohort. A valid WAT-1 result was available for 294 patients (94.5% of 311), who constitute the population in which the frequency of IWS is reported. IWS, defined as at least one WAT-1 score of 3 or higher during the postoperative pCICU stay, occurred in 136 of them (46.3%, 95% CI 40.6–52.0). A further 8 patients had a valid WAT-1 result but incomplete information on at least one of the ten variables of the principal model. Because all models were fitted as complete-case analyses, the multivariable analyses shown in Table 1 and Figure 1 are based on the remaining 286 patients (97.3% of 294), of whom 131 (45.8%, 95% CI 40.1–51.6) had IWS. Percentages given in each box refer to the box directly above it. CPB: cardiopulmonary bypass; IWS: iatrogenic withdrawal syndrome; pCICU: pediatric cardiac intensive care unit; WAT-1: Withdrawal Assessment Tool-1.

Supplementary Figure S2: Algorithm for perioperative analgesia and sedation. EEG: electroencephalogram; i.v.: intravenous; MAC: mean airway concentration; OTE: on-table extubation; pCICU: pediatric cardiac intensive care unit; p.o.: per os; TEE: transoesophageal echocardiography; WAT-1: Withdrawal Assessment Tool 1.

Supplementary Figure S3: WAT-1-guided sedation-weaning algorithm. Weaning protocol of the study center (institutional SOP 2.15.04.02, section 3.2.2, version 01, 1 August 2023). WAT-1 is assessed three times per 24 h; IWS is defined as a WAT-1 score ≥ 3. Infusion rates are tapered (score < 3), the tapering interval is prolonged to 24 h (score ≥ 3), or prolonged with reversal of the last reduction step score > 7). Redrawn and translated from the German-language original. IWS: iatrogenic withdrawal syndrome; WAT-1: Withdrawal Assessment Tool-1.

Supplementary Table S1: Sensitivity analysis: patients without any WAT-1 assessment (n = 17) compared with the complete-case population of the principal model (n = 286). CPBT: Cardiopulmonary bypass time; RACHS-1: Risk Adjustment for Congenital Heart Surgery; WAT-1: Withdrawal Assessment Tool 1.

Supplementary Table S2: Characteristics of the mechanically ventilated subgroup (n = 162) compared with the patients without any WAT-1 assessment (n = 17). CPBT: Cardiopulmonary bypass time; RACHS-1: Risk Adjustment for Congenital Heart Surgery; WAT-1: Withdrawal Assessment Tool 1.

Supplementary Figure S4: Exploratory subgroup analysis of the 77 patients with documented lactatemia and a valid WAT-1 result. Association of three lactatemia subtypes (low cardiac output (type A), metabolic derailment (type B), and lactatemia meeting neither criterion) with the occurrence of IWS. None of the estimates reached statistical significance. Odds ratios are shown with 95% confidence intervals and p-values. WAT-1: Withdrawal Assessment Tool-1; IWS: iatrogenic withdrawal syndrome.

Supplementary Figure S5: Exploratory subgroup analysis of the 49 patients with univentricular physiology. Association of the palliation stage with the occurrence of IWS, with biventricular repair as the reference category. None of the estimates reached statistical significance. Odds ratios are shown with 95% confidence intervals and p-values. IWS: iatrogenic withdrawal syndrome.

Supplementary Figure S1

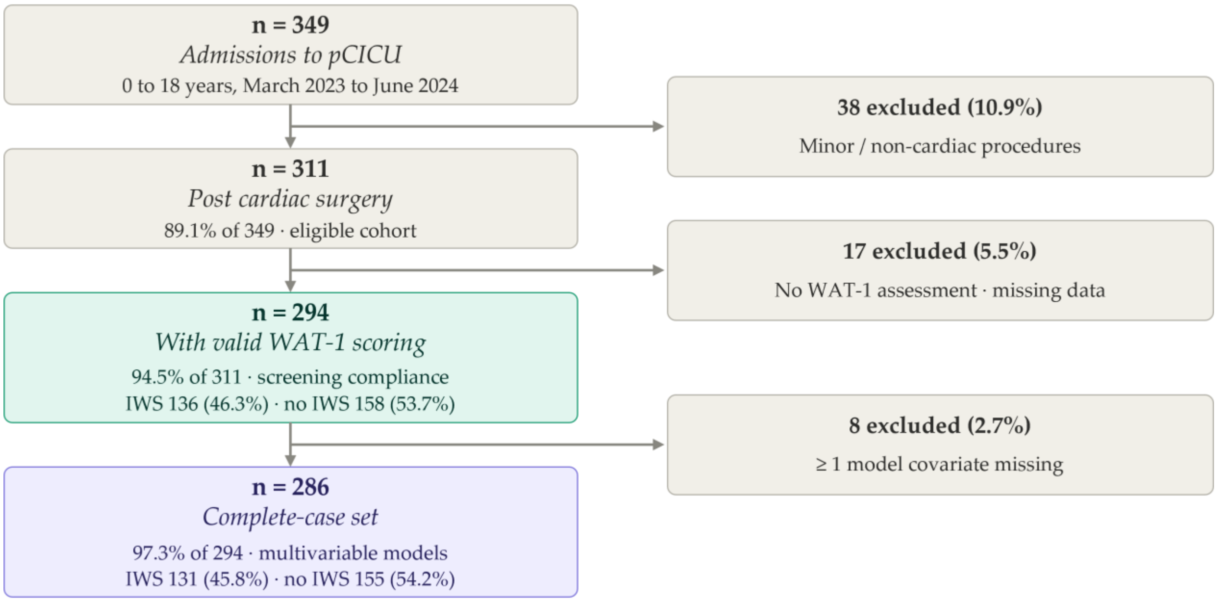

Supplementary Figure S2

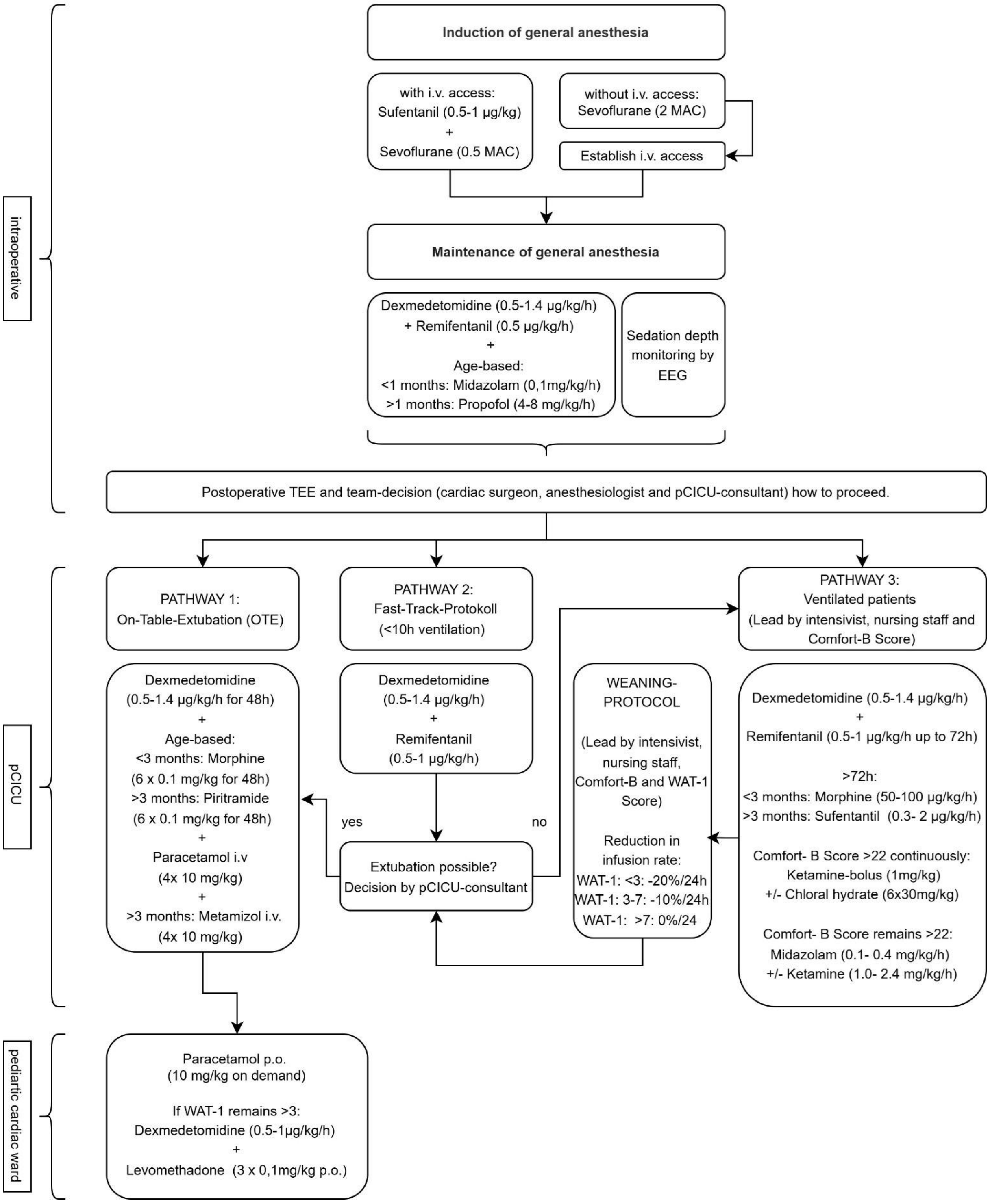

Supplementary Figure S3

Prevention, Diagnosis and Therapy of Pediatric Delirium and Iatrogenic Withdrawal Syndrome · Excerpt · Clinic-internal SOP

To guide the adequate treatment of IWS following diagnosis, the following scheme is applied according to the WAT-1 score:

Score < 3:

- Midazolam: reduction of the infusion rate by 20% every 12 h
- Morphine / Sufentanil: reduction of the infusion rate by 10% every 12 h
- Dexmedetomidine: only reduce once benzodiazepines and opiates have been terminated. Then reduction of the infusion rate by 0.3–0.5 μg/kg/min every 12–24 hours

Score ≥ 3:

Reduction interval of all substances mentioned extended to 24 h

Score > 7:

Reduction interval of all substances mentioned to be extended to 24 h and the last reduction step to be reversed

Score bands

Score < 3

Substance-specific reduction

Score ≥ 3

Reduction interval 24 h

Score > 7

Reduction interval 24 h + last step reversed

Excerpt from the clinic-internal SOP 2.15.04.02 "Prevention, Diagnosis and Therapy of Pediatric Delirium and Iatrogenic Withdrawal Syndrome", Version 01, as of 01.08.2023 — Section 3.2.2 · Universitäres Herz- und Gefäßzentrum Hamburg, PICU H2c · Internal document — not for external distribution.

Supplementary Table S1

Characteristics	No WAT-1 screening (%)	Complete-case population (%)	*p* Value
**n**	**17 (100.0)**	**286 (100.0)**	
Age			
Neonates (0–28 days)	5 (29.4)	58 (20.3)	
Infants (> 28 days–1 year)	8 (47.1)	116 (40.6)	0.434
Toddlers (> 1–6 years)	3 (17.6)	50 (17.5)	
School children and adolescents (> 6–18 years)	1 (5.9)	62 (21.7)	
RACHS-1			
1	3 (17.6)	34 (11.9)	
2	7 (41.2)	96 (33.6)	
3	3 (17.6)	106 (37.1)	0.598
4	2 (11.8)	34 (11.9)	
5	0 (0.0)	1 (0.3)	
6	2 (11.8)	15 (5.2)	
Univentricular physiology			
No	14 (82.4)	243 (85.0)	1.000
Yes	3 (17.6)	43 (15.0)	
CPBT (minutes)			
0–30	3 (17.6)	45 (15.7)	
> 30–60	1 (5.9)	49 (17.1)	0.357
> 60–90	7 (41.2)	80 (28.0)	
> 90–120	1 (5.9)	54 (18.9)	
> 120	5 (29.4)	58 (20.3)	
Metabolic derailment			
No	10 (58.8)	178 (62.2)	0.980
Yes	7 (41.2)	108 (37.8)	
Postoperative desaturation			
No	13 (76.5)	241 (84.3)	0.611
Yes	4 (23.5)	45 (15.7)	
Lactatemia			
No	11 (64.7)	215 (75.2)	0.499
Yes	6 (35.3)	71 (24.8)	
Fever			
No	12 (70.6)	222 (77.6)	0.708
Yes	5 (29.4)	64 (22.4)	
Suspected infection			
No	14 (82.4)	258 (90.2)	0.531
Yes	3 (17.6)	28 (9.8)	
Hydrocortisone			
No	9 (52.9)	194 (67.8)	0.316
Yes	8 (47.1)	92 (32.2)	
Duration of mechanical ventilation (hours)			
On-table extubation	2 (11.8)	121 (42.3)	
Fast-track extubation (0–10)	3 (17.6)	48 (16.8)	0.030
Short-term ventilation (10–100)	5 (29.4)	68 (23.8)	
Long-term ventilation (> 100)	7 (41.2)	49 (17.1)	

Supplementary Table S2

Characteristics	No WAT-1 screening (%)	Ventilated subgroup (%)	*p* Value
**n**	**17 (100.0)**	**162 (100.0)**	
Age			
Neonates (0–28 days)	5 (29.4)	48 (29.6)	
Infants (> 28 days–1 year)	8 (47.1)	74 (45.7)	0.828
Toddlers (> 1–6 years)	3 (17.6)	20 (12.3)	
School children and adolescents (> 6–18 years)	1 (5.9)	20 (12.3)	
RACHS-1			
1	3 (17.6)	9 (5.6)	
2	7 (41.2)	55 (34.0)	
3	3 (17.6)	57 (35.2)	0.222
4	2 (11.8)	29 (17.9)	
5	0 (0.0)	0 (0.0)	
6	2 (11.8)	12 (7.4)	
Univentricular physiology			
No	14 (82.4)	134 (82.7)	1.000
Yes	3 (17.6)	28 (17.3)	
CPBT (minutes)			
0–30	3 (17.6)	23 (14.2)	
> 30–60	1 (5.9)	20 (12.3)	
> 60–90	7 (41.2)	41 (25.3)	0.444
> 90–120	1 (5.9)	32 (19.8)	
> 120	5 (29.4)	46 (28.4)	
Metabolic derailment			
No	10 (58.8)	74 (45.7)	0.437
Yes	7 (41.2)	88 (54.3)	
Postoperative desaturation			
No	13 (76.5)	126 (77.8)	1.000
Yes	4 (23.5)	36 (22.2)	
Lactatemia			
No	11 (64.7)	108 (66.7)	1.000
Yes	6 (35.3)	54 (33.3)	
Fever			
No	12 (70.6)	119 (73.5)	1.000
Yes	5 (29.4)	43 (26.5)	
Suspected infection			
No	14 (82.4)	139 (85.8)	0.982
Yes	3 (17.6)	23 (14.2)	
Hydrocortisone			
No	9 (52.9)	80 (49.4)	0.981
Yes	8 (47.1)	82 (50.6)	
Duration of mechanical ventilation (hours)			
On-table extubation	2 (11.8)	0 (0.0)	
Fast-track extubation (0–10)	3 (17.6)	46 (28.4)	< 0.001
Short-term ventilation (10–100)	5 (29.4)	67 (41.4)	
Long-term ventilation (> 100)	7 (41.2)	49 (30.2)	

Supplementary Figure S4

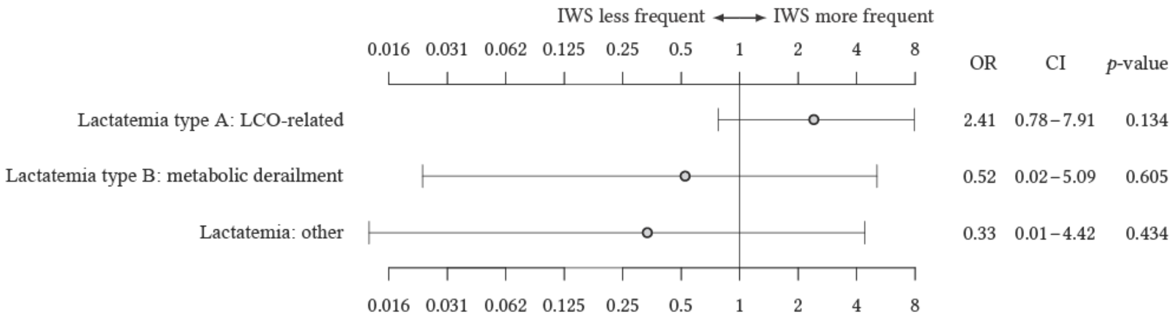

Supplementary Figure S5

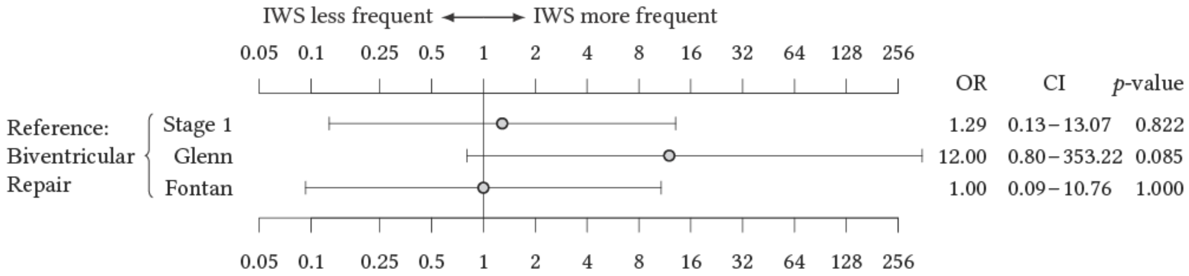

Supplement: Supplementary file 1 [file children-13-01307-s001.zip › children-4474870-supplementary.pdf]
